# Pan-cancer analysis of genomic and transcriptomic data reveals the prognostic relevance of human proteasome genes in different cancer types

**DOI:** 10.1186/s12885-022-10079-4

**Published:** 2022-09-19

**Authors:** Peter Larsson, Daniella Pettersson, Hanna Engqvist, Elisabeth Werner Rönnerman, Eva Forssell-Aronsson, Anikó Kovács, Per Karlsson, Khalil Helou, Toshima Z. Parris

**Affiliations:** 1 Department of Oncology, Institute of Clinical Sciences, Sahlgrenska Academy, University of Gothenburg, Gothenburg, Sweden; 2 Sahlgrenska Center for Cancer Research, Sahlgrenska Academy, University of Gothenburg, Gothenburg, Sweden; 3 Department of Clinical Pathology, Sahlgrenska University Hospital, Gothenburg, Sweden; 4 Department of Medical Radiation Sciences, Institute of Clinical Sciences, Sahlgrenska Academy, University of Gothenburg, Gothenburg, Sweden; 5 Department of Medical Physics and Biomedical Engineering, Sahlgrenska University Hospital, Gothenburg, Sweden; 6 Department of Oncology, Sahlgrenska University Hospital, Gothenburg, Sweden

**Keywords:** Proteasome gene family, Gene expression profiling, Prognosis, Cancer, Prognostic biomarkers

## Abstract

**Background:**

The human proteasome gene family (PSM) consists of 49 genes that play a crucial role in cancer proteostasis. However, little is known about the effect of PSM gene expression and genetic alterations on clinical outcome in different cancer forms.

**Methods:**

Here, we performed a comprehensive pan-cancer analysis of genetic alterations in PSM genes and the subsequent prognostic value of PSM expression using data from The Cancer Genome Atlas (TCGA) containing over 10,000 samples representing up to 33 different cancer types. External validation was performed using a breast cancer cohort and KM plotter with four cancer types.

**Results:**

The PSM genetic alteration frequency was high in certain cancer types (*e.g.* 67%; esophageal adenocarcinoma), with DNA amplification being most common. Compared with normal tissue, most PSM genes were predominantly overexpressed in cancer. Survival analysis also established a relationship with PSM gene expression and adverse clinical outcome, where *PSMA1* and *PSMD11* expression were linked to more unfavorable prognosis in ≥ 30% of cancer types for both overall survival (OS) and relapse-free interval (PFI). Interestingly, *PSMB5* gene expression was associated with OS (36%) and PFI (27%), and OS for *PSMD2* (42%), especially when overexpressed.

**Conclusion:**

These findings indicate that several PSM genes may potentially be prognostic biomarkers and novel therapeutic targets for different cancer forms.

**Supplementary Information:**

The online version contains supplementary material available at 10.1186/s12885-022-10079-4.

## Introduction

In eukaryotic cells, about 80% of intracellular protein degradation is mediated via the nonlysosomal ubiquitin–proteasome system (UPS) [1–3]. The 26S proteasome (2500 kDa) is an evolutionarily conserved protein complex that uses proteolysis to selectively degrade damaged and misfolded polyubiquitinated proteins [4–6]. The 26S proteasome complex consists of one or two 19S regulatory particles (900 kDa) that recognize, deubiquitinate, and translocate protein substrates to the barrel-shaped 20S protein core (700 kDa) where protein substrates are cleaved into smaller oligopeptides (< 25 amino acids) [7]. The 20S core particle consists of four stacked heteroheptameric rings (α_1–7_ β_1–7_ β_1–7_ α_1–7_) with two highly conserved outer α rings (serve as a gate to restrict access to the catalytic core) and two inner β rings (only 3/7 β subunits are proteolytically active, namely β1 (caspase-like), β2 (trypsin-like), and β5 (chymotrypsin-like)) [3, 5, 6, 8, 9]. In normal cells, proteasome abundance is regulated by controlling the expression of proteasome subunits and assembly chaperones [3]. Furthermore, proteasome abundance and proteolytic activity have been found to be dependent on tissue type and age [5, 10, 11].

The proteasome gene family (PSM) consists of 49 genes, including subunits for the 20S α and β rings (*n* = 19; class I), 26S ATPases and non-ATPases (*n* = 20; class II), proteasome activators and a PSMC3 interacting protein (*n* = 5; class III), a proteasome inhibitor subunit (class IV), and proteasome assembly chaperones (*n* = 4; class V) [4–6, 8, 12, 13]. Consequently, tissue-specific proteasomes have been identified in lymphoid and non-lymphoid tissues that are induced by interferon-γ (immunoproteasome containing β1i (*PSMB9*), β2i (*PSMB10*), and β5i (*PSMB8*) instead of constitutive β1 (*PSMB6*), β2 (*PSMB7*), and β5 (*PSMB5*) subunits), thymic epithelial cells (thymoproteasome containing β5t (*PSMB11*) instead of β5), and the testes during spermatogenesis (spermatoproteasome containing α4s (*PSMA8*) instead of α4 (*PSMA7*)) [14–17]. Dysfunction of the proteasome has been associated with neurodegenerative diseases, aging, and cancer [18–21]. Subsequent downregulation of the 26S proteasome in certain cells, *e.g.* cancer stem cells, has led to the development of pharmaceutical agents to counteract proteasome dysfunction by stimulating 26S proteasome activity [22–25]. Genetic aberrations in the *PSMB8* immunoproteasome gene have been associated with cancer and a wide range of immune and inflammatory diseases, *e.g.* Nakajo-Nishimura syndrome, CANDLE syndrome, and intestinal M. tuberculosis infection [11, 15]. Additionally, other PSM genes have been associated with cancer progression (*e.g. PSMD9* (*p27*) and *PSMD10* (*p28*)), increased radiation sensitivity in breast cancer (*e.g.* absence of *p27*), as well as, increased risk of colorectal cancer (*e.g. PSMB8* and *PSMB9*) [3, 11, 26–29]. Mutations in other PSM genes (*e.g.* A20T, A27P, C63Y, and M45I in the *PSMB5* gene) have also been reported to cause resistance to certain proteasome inhibitors [30, 31].

Although proteasome inhibitors were initially developed to prevent cancer-related cachexia, the abnormally high proteasome activity observed in human cancer cells has thus led to the proteasome becoming an attractive target for anticancer drug development [7, 32]. In cancer cells, proteasome abundance is controlled by the *NRF1* and *NRF2* transcription factors, which in turn promotes resistance to environmental stresses, as well as, chemo- and radiation therapy [3, 23, 33–37]. The first clinically used proteasome inhibitor, bortezomib (brand name Velcade®), was approved by the Food and Drug Association in 2003 as a salvage treatment with dexamethasone for relapsed refractory multiple myeloma [2]. Subsequent side effects and problems with bortezomib-based therapy resistance resulted in the development of second-generation inhibitors such as carfilzomib, ixazomib, delanzomib, marizomib, and oprozomib [2, 32, 37]. With the exception of ixazomib, the majority of proteasome inhibitors bind to the β5 subunit at relatively low concentrations, and the β1 and β2 subunits at higher concentrations. However, recent studies have shown that β5/β2 or β5/β1 co-inhibition provides a significantly improved effect [38, 39].

Although proteasome inhibitor-based cancer treatments have been used for about 20 years, their clinical utility for various cancer types has yet to be elucidated, in part due to our limited understanding of PSM gene expression in different cancer forms. Here, we identified genetic alterations and aberrant transcriptomic patterns in PSM genes across 33 cancer forms to delineate their effect on prognosis, thereby identifying cancer forms that may benefit from proteasome inhibitor-based treatment.

## Methods

### Patient cohorts and data acquisition

**Fig. 1:**
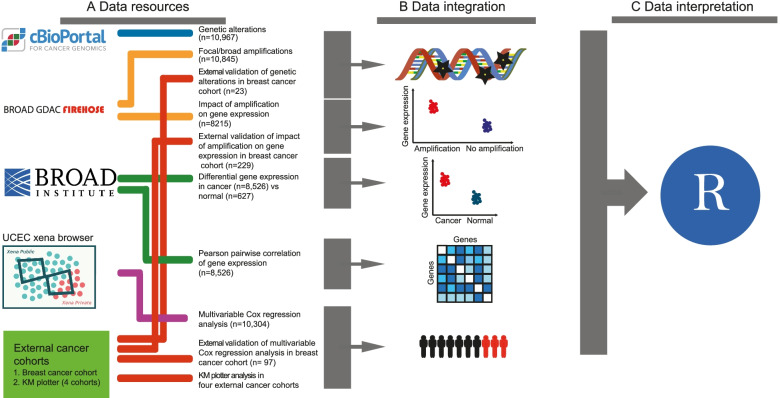
Flowchart depicting the study design and workflow. **A** Genomic and transcriptomic data were collected from multiple sources and validated with a breast cancer cohort and KM plotter. **B** Both interactive tools and collected data were used to determine genomic alterations and its effect on gene expression. Furthermore, differential expression between cancer and normal tissue, co-expressed genes was determined, and how this affects cancer patient survival. **C** We used the statistical tool R to perform statistical calculations and to generate figures

A comprehensive genomic and transcriptomic analysis of the PSM gene family (Table 1) was performed using The Cancer Genome Atlas (TCGA) pan-cancer dataset comprised of close to 11,000 primary and/or metastatic tumor samples corresponding to 33 cancer types and 11 pan-organ systems (*i.e.* central nervous system (CNS), endocrine, gastrointestinal, gynecologic, head and neck, hematologic and lymphatic malignancies, melanocytic, neural-crest derived, soft tissue, thoracic, urologic), as previously described [40]. The patient cohorts are described in detail in Table 2; SKCM and THCA contain data for primary and metastatic samples. First, genomic profiling data were retrieved from the interactive web-based cBioPortal tool [41] to assess the genomic alteration frequency in the PSM genes for 10,967 TCGA tumor samples corresponding to 10,953 patients (30 cancer types representing 10 pan-cancer organ systems). Focal and arm-level (henceforth termed broad) amplification regions in each cancer type were identified using copy number GISTIC2 data (focal amplifications and arm-level significance; Supplementary Table 1) from Broad GDAC Firehose [42], followed by an evaluation of the impact of DNA amplification on gene expression patterns using UNC RNASeqV2 level 3 expression (normalized RSEM; mRNA). A list of consensus cancer driver genes and cancer drivers associated with DNA amplification were compiled from previously published lists [43, 44]. Of the genetic variants identified in cBioPortal, fusions, missense, nonsense, frameshift deletion/insertion, inframe deletion/insertion, translation start site, and nonstop mutations were classified as potentially deleterious variants (*i.e.* mutations with a functional impact due to amino acid changes). Furthermore, functionally important deleterious variants were classified as SIFT score 0–0.05 (deleterious) and/or Polyphen-2 score 0.453–1 (probably/possibly damaging). Second, gene expression analysis was performed using UNC RNASeqV2 level 3 expression (normalized RSEM; mRNA) retrieved from Broad GDAC Firehose for 8,526 tumor specimens (corresponding to 33 cancer types) and 627 corresponding normal specimens from the TCGA consortium. Lastly, multivariable Cox regression analysis was performed using log2 FPKM gene expression data and clinical data retrieved from UCSC Xena Browser and Genomic Data Commons (GDC) Supplemental Table S1 [45, 46] for 10,304 GDC TCGA samples (corresponding to 33 cancer types). PSM gene expression was categorized from RNA sequencing data (FPKM log2) as low expression (lower than median expression, FPKM log2 4.398046) and high expression (higher than median expression) by calculating the quantiles (0, 25, 50, 75, 100%) for the 49 PSM genes. Hazard ratios (HR) < 1 depicts reduced risk at high expression levels, while HR > 1 illustrates increased risk at high expression. The study flowchart is shown in Fig. 1.

**Table 1:** The 49 human proteasome gene family members (proteasome subunits and proteasome-interacting proteins)

**Gene symbol and full name**		**Subunit**^**a**^	**Chromosome**^**b**^	**Aliases**^**c**^	**UniProtKB accession**^**c**^	**Sequence length**^**c**^ (amino acids)	**MW**^**c**^ (Da)
***Class I: Proteasome 20S subunit***							
* PSMA1*	Proteasome 20S subunit alpha 1	α6	11p15.2	HC2, NU, PROS30, PSC2	P25786	263	29,556
* PSMA2*	Proteasome 20S subunit alpha 2	α2	7p14.1	HC3, PSC3	P25787	234	25,899
* PSMA3*	Proteasome 20S subunit alpha 3	α7	14q23.1	HC8, PSC8	P25788	255	28,433
* PSMA4*	Proteasome 20S subunit alpha 4	α3	15q25.1	HC9, PSC9	P25789	261	29,484
* PSMA5*	Proteasome 20S subunit alpha 5	α5	1p13.3	ZETA	P28066	241	26,411
* PSMA6*	Proteasome 20S subunit alpha 6	α1	14q13.2	PROS27	P60900	246	27,399
* PSMA7*	Proteasome 20S subunit alpha 7	α4	20q13.33	HSPC	O14818	248	27,887
* PSMA8*	Proteasome 20S subunit alpha 8	-	18q11.2	PSMA7L	Q8TAA3	256	28,530
* PSMB1*	Proteasome 20S subunit beta 1	β6	6q27	PSC5	P20618	241	26,489
* PSMB2*	Proteasome 20S subunit beta 2	β4	1p34.3	HC7-I	P49721	201	22,836
* PSMB3*	Proteasome 20S subunit beta 3	β3	17q12	HC10-II, MGC4147	P49720	205	22,949
* PSMB4*	Proteasome 20S subunit beta 4	β7	1q21.3	PROS26	P28070	264	29,204
* PSMB5*	Proteasome 20S subunit beta 5	β5	14q11.2	LMPX, MB1, X	P28074	263	28,480
* PSMB6*	Proteasome 20S subunit beta 6	β1	17p13.2	LMPY, Y	P28072	239	25,358
* PSMB7*	Proteasome 20S subunit beta 7	β2	9q33.3	Z	Q99436	277	29,965
* PSMB8*	Proteasome 20S subunit beta 8	β5i	6p21.32	LMP7, PSMB5i, RING10, Y2	P28062	276	30,354
* PSMB9*	Proteasome 20S subunit beta 9	β1i	6p21.32	LMP2, PSMB6i, RING12	P28065	219	23,264
* PSMB10*	Proteasome 20S subunit beta 10	β2i	16q22.1	LMP10, MECL1	P40306	273	28,936
* PSMB11*	Proteasome 20S subunit beta 11	β5t	14q11.2		A5LHX3	300	32,530
***Class II: Proteasome 26S subunit***							
* PSMC1*	Proteasome 26S subunit, ATPase 1	Rpt2	14q32.11	S4, p56	P62191	440	49,185
* PSMC2*	Proteasome 26S subunit, ATPase 2	Rpt1	7q22.1	MSS1	P35998	433	48,634
* PSMC3IP*	PSMC3 Interacting Protein		17q21.2	HOP2, TBPIP	Q9P2W1	217	24,906
* PSMC3*	Proteasome 26S subunit, ATPase 3	Rpt5	11p11.2	TBP1	P17980	439	49,204
* PSMC4*	Proteasome 26S subunit, ATPase 4	Rpt3	19q13.2	TBP-7	P43686	418	47,366
* PSMC5*	Proteasome 26S subunit, ATPase 5	Rpt6	17q23.3	SUG1	P62195	406	45,626
* PSMC6*	Proteasome 26S subunit, ATPase 6	Rpt4	14q22.1	SUG2	P62333	389	44,173
* PSMD1*	Proteasome 26S subunit, non-ATPase 1	Rpn2	2q37.1	S1, P112, Rpn2	Q99460	953	105,836
* PSMD2*	Proteasome 26S subunit, non-ATPase 2	Rpn1	3q27.1	TRAP2	Q13200	908	100,200
* PSMD3*	Proteasome 26S subunit, non-ATPase 3	Rpn3	17q21.1	S3, P58, Rpn3	O43242	534	60,978
* PSMD4*	Proteasome 26S subunit, non-ATPase 4	Rpn10	1q21.3	MCB1	P55036	377	40,737
* PSMD5*	Proteasome 26S subunit, non-ATPase 5	-	9q33.2	KIAA0072	Q16401	504	56,196
* PSMD6*	Proteasome 26S subunit, non-ATPase 6	Rpn7	3p14.1	KIAA0107, PFAAP4	Q15008	389	45,531
* PSMD7*	Proteasome 26S subunit, non-ATPase 7	Rpn8	16q23.1	MOV34L	P51665	324	37,025
* PSMD8*	Proteasome 26S subunit, non-ATPase 8	Rpn12	19q13.2	S14, Nin1p, p31, HIP6, HYPF, Rpn12	P48556	350	39,612
* PSMD9*	Proteasome 26S subunit, non-ATPase 9	-	12q24.31	p27, Rpn4	O00233	223	24,682
* PSMD10*	Proteasome 26S subunit, non-ATPase 10	Gankyrin	Xq22.3	p28	O75832	226	24,428
* PSMD11*	Proteasome 26S subunit, non-ATPase 11	Rpn6	17q11.2	S9, p44.5, MGC3844, Rpn6	O00231	422	47,464
* PSMD12*	Proteasome 26S subunit, non-ATPase 12	Rpn5	17q24.2	p55, Rpn5	O00232	456	52,904
* PSMD13*	Proteasome 26S subunit, non-ATPase 13	Rpn9	11p15.5	p40.5, Rpn9	Q9UNM6	376	42,945
* PSMD14*	Proteasome 26S subunit, non-ATPase 14	Rpn11	2q24.2	POH1	O00487	310	34,577
***Class III: Proteasome activator***							
* PSME1*	Proteasome activator subunit 1	PA28α	14q12	IFI5111	Q06323	249	28,723
* PSME2*	Proteasome activator subunit 2	PA28β	14q12	PA28beta	Q9UL46	239	27,402
* PSME3*	Proteasome activator subunit 3	PA28γ	17q21.31	Ki, PA28-gamma, REG-GAMMA, PA28G	P61289	254	29,506
* PSME4*	Proteasome activator subunit 4	PA200	2p16.2	KIAA0077	Q14997	1,843	211,334
***Class IV: Proteasome inhibitor***							
* PSMF1*	Proteasome inhibitor subunit 1	PI31	20p13	PI31	Q92530	271	29,817
***Class V: Proteasome assembly chaperone***							
* PSMG1*	Proteasome Assembly Chaperone 1		21q22.2	C21LRP, DSCR2, PAC1	O95456	288	32,854
* PSMG2*	Proteasome Assembly Chaperone 2		18p11.21	HCCA3, PAC2, TNFSF5IP1	Q969U7	264	29,396
* PSMG3*	Proteasome Assembly Chaperone 3		7p22.3	C7orf48, PAC3	Q9BT73	122	13,104
* PSMG4*	Proteasome Assembly Chaperone 4		6p25.2	C6orf86, PAC4	Q5JS54	123	13,775

Data obtained from ^a ^Gomes AV et al*.,* ^b ^Genome Reference Consortium Human GRCh38.p12/hg38, ^c ^UniProtKB

**Table 2:** TCGA cancer types and corresponding pan-cancer organ systems

Disease name and pan-organ system	Cohort	RNA-seq data^a^		Survival analysis^b^	cBioPortal^c^	KM plotter^d^
		**Cancer tissue**	**Normal tissue**			
***Central nervous system (CNS)***						
Glioblastoma multiforme	GBM	166	5	167	592	
Brain lower grade glioma	LGG	530	0	528	514	
***Endocrine***						
Adrenocortical carcinoma	ACC	79	0	79	92	
Thyroid carcinoma	THCA	496	58	510	500	
***Gastrointestinal***						
Cholangiocarcinoma	CHOL	36	9	36	36	
Colon adenocarcinoma	COAD	191	0	469		
Esophageal carcinoma	ESCA^e^	185	11	162	182	
Liver hepatocellular carcinoma	LIHC	147	50	374	372	364
Pancreatic adenocarcinoma	PAAD	56	0	178	184	
Rectum adenocarcinoma	READ	72	0	166		
Colorectal adenocarcinoma/Rectum adenocarcinoma	COADREAD^f^		0		594	
Stomach adenocarcinoma	STAD	415	35	375	440	875
***Gynecologic***						
Breast invasive carcinoma	BRCA	1026	108	1103	1084	1879
Cervical and endocervical cancers	CESC	159	0	306	297	
Ovarian serous cystadenocarcinoma	OV	265	0	379	585	1656
Uterine corpus endometrial carcinoma	UCEC	369	0	548	529	
***Head and neck***						
Head and neck squamous cell carcinoma	HNSC	425	42	502	523	
***Hematologic and lymphatic malignancies***						
Lymphoid neoplasm diffuse large B-cell lymphoma	DLBC	48	0	48	48	
Acute myeloid leukemia	LAML	173	0	151	200	
Thymoma	THYM	120	0	119	123	
***Melanocytic***						
Skin cutaneous melanoma	SKCM	472	0	471	448	
Uveal melanoma	UVM	80	0	80	80	
***Neural crest-derived***						
Pheochromocytoma and paraganglioma	PCPG	184	3	183	178	
***Soft tissue***						
Sarcoma	SARC	105	0	263	255	
Uterine carcinosarcoma	UCS	57	0	56	57	
***Thoracic***						
Lung adenocarcinoma	LUAD	490	58	526	566	1925
Lung squamous cell carcinoma	LUSC	482	50	501	487	
Mesothelioma	MESO	87	0	86	87	
***Urologic***						
Bladder urothelial carcinoma	BLCA	223	19	411	411	
Kidney chromophobe	KICH	66	25	65	65	
Kidney renal clear cell carcinoma	KIRC	507	72	535	512	
Kidney renal papillary cell carcinoma	KIRP	161	30	289	283	
Prostate adenocarcinoma	PRAD	498	52	499	494	
Testicular germ cell tumors	TGCT	156	0	139	149	
Total		8526	627	10,304	10,967	6699

^a ^UNC RNASeqV2 level 3 expression (normalized RSEM) data were retrieved from Broad GDAC Firehose (https://gdac.broadinstitute.org/)

^b ^Survival analysis was performed using the dataset https://gdc.cancer.gov/about-data/publications/PanCan-Clinical-2018

^c ^Mutational profiling data (mutated genes, CNA genes, and fusion genes) was retrieved from cBioPortal for Cancer Genomics, http://www.cbioportal.org/study/summary?id=laml_tcga_pan_can_atlas_2018%2Cacc_tcga_pan_can_atlas_2018%2Cblca_tcga_pan_can_atlas_2018%2Clgg_tcga_pan_can_atlas_2018%2Cbrca_tcga_pan_can_atlas_2018%2Ccesc_tcga_pan_can_atlas_2018%2Cchol_tcga_pan_can_atlas_2018%2Ccoadread_tcga_pan_can_atlas_2018%2Cdlbc_tcga_pan_can_atlas_2018%2Cesca_tcga_pan_can_atlas_2018%2Cgbm_tcga_pan_can_atlas_2018%2Chnsc_tcga_pan_can_atlas_2018%2Ckich_tcga_pan_can_atlas_2018%2Ckirc_tcga_pan_can_atlas_2018%2Ckirp_tcga_pan_can_atlas_2018%2Clihc_tcga_pan_can_atlas_2018%2Cluad_tcga_pan_can_atlas_2018%2Clusc_tcga_pan_can_atlas_2018%2Cmeso_tcga_pan_can_atlas_2018%2Cov_tcga_pan_can_atlas_2018%2Cpaad_tcga_pan_can_atlas_2018%2Cpcpg_tcga_pan_can_atlas_2018%2Cprad_tcga_pan_can_atlas_2018%2Csarc_tcga_pan_can_atlas_2018%2Cskcm_tcga_pan_can_atlas_2018%2Cstad_tcga_pan_can_atlas_2018%2Ctgct_tcga_pan_can_atlas_2018%2Cthym_tcga_pan_can_atlas_2018%2Cthca_tcga_pan_can_atlas_2018%2Cucs_tcga_pan_can_atlas_2018%2Cucec_tcga_pan_can_atlas_2018%2Cuvm_tcga_pan_can_atlas_2018

^d ^Survival analysis using KM plotter, https://kmplot.com/analysis/

^e ^Esophageal adenocarcinoma and Esophageal squamous carcinoma was merged into one as Esophageal carcinoma

^f ^COAD and READ was merged in cBioPortal dataset

### External validation

To validate our findings, we re-evaluated genomic profiling data (array comparative genomic hybridization, SNP genotyping, RNA-seq) [47, 48] from 229 breast invasive carcinomas. Mutation signatures for the PSM genes were determined for 23 of the 229 samples (Supplementary Table 2), CNA in all samples (Supplementary Table 3), and correlation between individual PSM mRNA expression and overall survival (OS; defined as the time from initial diagnosis to death of any cause) using both univariable and multivariable analysis (adjusted for age and tumor grade). KM plotter [49] was used to validate the correlation between individual PSM mRNA expression and OS in gastric- (RNA microarray), breast- (RNA microarray), lung- (RNA microarray), ovarian- (RNA microarray), and liver cancer (RNA-seq). For each gene, the following settings were selected in KM plotter: (1) Split patients by: ‘median’ expression, (2) Survival: OS, and (3) Probe options: user selected probe set. Multipletesting.com was then used to calculate the False Discovery Rate (FDR) set to 5% [50]. All procedures were done in accordance with the Declaration of Helsinki and approved by the Medical Faculty Research Ethics Committee (Gothenburg, Sweden).

### Statistical analysis

*P* < 0.05 (two-sided) was considered to be statistically significant in R/Bioconductor (version 3.6.1). Hierarchical clustering of the log2-tranformed relative RNA-seq data (cancer vs mean normal samples) was performed with the pheatmap R package (version 1.0.12) [51] using the Manhattan distance metric and Ward’s minimum variance method (Ward.D2). The biological significance of DNA amplification was evaluated by comparing the gene expression patterns between PSM genes showing amplification (classified as AMP in cBioPortal) and no amplification (classified as no alteration or all other mutation types in cBioPortal). To compare gene expression levels between cancer and normal samples, cancer types with no available normal samples (ACC, CESC, COAD, DLBC, LAML, LGG, MESO, OV, PAAD, READ, SARC, SKCM, TGCT, THYM, UCEC, UCS, UVM) were removed. Then, box plots were constructed using the ggpubr (version 0.2.4.999) [52] and rstatix (version 0.4.0.999) [53] R packages with the Wilcoxon test and Benjamini–Hochberg adjusted p-values (ns = not significant (*P* > 0.05); *P < 0.05; **P ≤ 0.01; ***P ≤ 0.001; ****P ≤ 0.0001). The pairwise Pearson's correlation coefficient (*r*) (0 < *r* < 0.4 (weak); 0.4 < *r* < 0.7 (moderate); *r* > 0.7 (strong)) was calculated per gene pair using the basic stats R package to determine the level of co-expression. Gene expression correlation matrices were visualized using the corrplot R package (version 0.84) [54] with Ward D2 hierarchical clustering and *P* < 0.05 (95% confidence intervals; 95% CI). As GDC deemed OS and progression-free interval (PFI; defined as life span during and after treatment without worsening disease) to be relatively accurate clinical outcome endpoints with little missing data, they were recommended for use in survival analyses. Therefore, multivariable Cox proportional hazard models were calculated for the 49 PSM genes using OS or PFI adjusted for available established prognostic markers (age and/or tumor grade). Forest plots were used to display HR for the effect of gene expression on OS or PFI with the forestplot R package (version 1.9) [55].

## Results

### Pan-cancer genomic profiling demonstrates prevalent DNA amplification of PSM genes

**Fig. 2:**
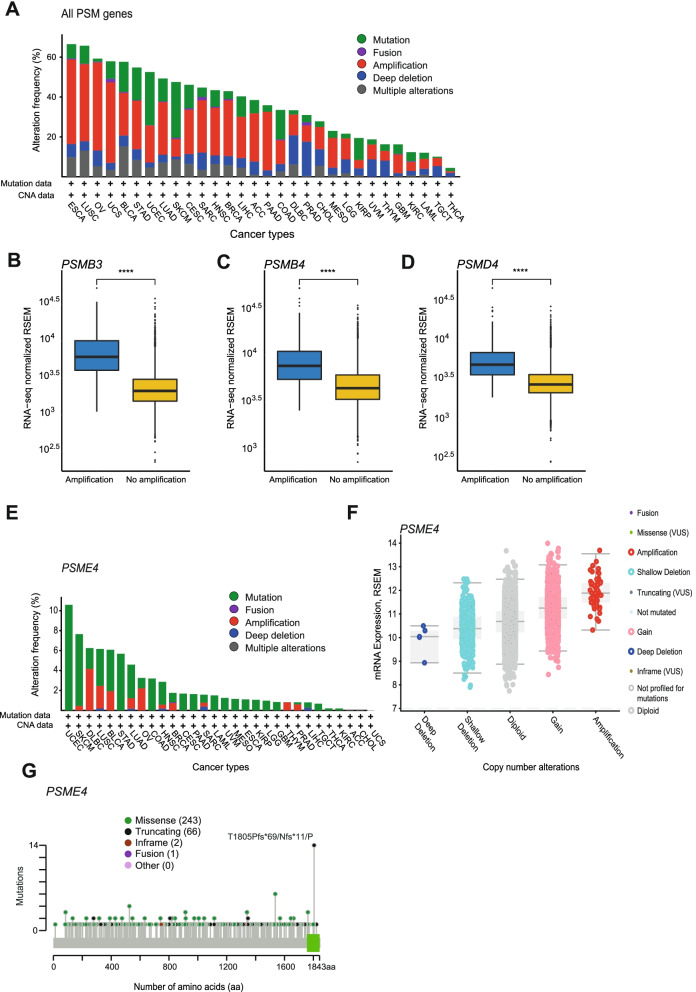
Bar charts depicting alteration frequency for the 49 PSM genes by cancer type using the interactive web-based online tool cBioPortal (cbioportal.org). **A** DNA amplification was shown to be prevalent in most cancer types, with ESCA and THCA showing the highest och lowest alteration frequencies, respectively. Box plots visualizing DNA amplification of (**B**) *PSMB3*, (**C**) *PSMB4*, (**D**) *PSMD4* and their effect on expression (RSEM). Wilcoxon test was used to calculate statistical significance (Benjamini–Hochberg adjusted p-values), ns = not significant (*P* ≥ 0.05); *P < 0.05; **P ≤ 0.01; ***P ≤ 0.001; ****P ≤ 0.0001. **E** *PSME4* gene was the most mutated of all PSM genes. Most *PSME4* mutations was found in the UCEC cancer type, where missense mutations were prevalent. **F** Beeswarm plot visualizing copy number alterations (CNA) and other types of mutations, and their effect on expression was generated in cBioPortal. Deep deletions in *PSME4* resulted in significantly lower expression. **G** Lollipop plot depicting the number of mutations across the *PSME4* gene. Missense mutations were prevalent (243 of 312 mutations), with a domain with unknown function containing 14 mutations (10 frameshift deletions in T1805Pfs*69, three frameshift insertions in T1805Nfs*11, and one missense in *T1805P*)

To assess the distribution of genetic alterations (*e.g.* inframe mutation, missense mutation, nonsense mutation, fusion, amplification, and nonstop mutation) in PSM genes in different cancer types, we used genomic profiling data retrieved from the web-based cBioPortal tool for over 10,000 tumor samples (representing 33 cancer types and 11 pan-cancer body groups) from the TCGA dataset (Tables 1 and 2). PSM genes were shown to be altered in approximately 67% of esophageal carcinoma (ESCA) cases (*n* = 182) and 66% of lung squamous cell carcinomas (LUSC, n = 487), but only 4% of thyroid carcinoma (THCA) cases (*n* = 500; Fig. 2A). Genetic alterations (predominantly DNA amplification) were subsequently detected in all PSM genes, with the vast majority of aberrations found in the *PSMD2* (6% of patient samples), *PSMB4* (4%), and *PSMD4* (4%) genes. In contrast, relatively few samples were found to harbor mutations in the *PSMA3* gene (approximately 1%; Supplementary Fig. 1). Interestingly, genetic aberrations in *PSMD2* were most frequently found in LUSC (37% of 487 cases).

GISTIC2 data from Broad GDAC Firehose were then used to evaluate the effect of DNA amplification of the 49 PSM genes on gene expression (Supplementary Table 1). Broad amplification of whole chromosome arms (p and q arms) was most prevalent in the different cancer types (mean ± SEM, 7.3 ± 0.9; range, 1–22), while focal amplification was found in 1.7 ± 0.4 (range, 0–12) cancer types per PSM gene. Furthermore, similar DNA amplification profiles were found for 10 PSM genes located on the same cytoband (*PSMB5* and *PSMB11*, 14q11.2; *PSME1* and *PSME2*, 14q12; *PSMC4* and *PSMD8*, 19q13.2; *PSMB4* and *PSMD4*, 1q21.3; *PSMB8* and *PSMB9*, 6p21.32; Supplementary Fig. 2) and a number of consensus cancer driver genes (e.g. *PSMB3* and *ERBB2*, 17q12; *PSME3* and *BRCA1*, 17q21.31) [43, 44]. Moreover, several PSM genes (*PSMA6-8*, *PSMB3-4*, *PSMB8-9*, *PSMC2*, *PSMC4-5*, *PSMD2-4*, *PSMD8*, *PSMD12*, and *PSMG3-4*) were amplified > 100 times across cancer types. Of these, *PSMB4* (1q21.3) and *PSMD4* (1q21.3) genes were amplified > 400 times, while *PSMD2* (3q27.1) was amplified almost 600 times. In general, DNA amplification was most prevalent in the BLCA (urologic), BRCA (gynecologic), LUSC (thoracic), LUAD (thoracic), OV (gynecologic), and UCEC (gynecologic) cancer types. DNA amplification events (broad and focal) resulted in significantly elevated RNA levels for all 49 PSM genes in amplified samples compared to non-amplified samples (*P* adjusted < 0.05; Supplementary Table 1), including *PSMB4* (1q21.3), *PSMD4* (1q21.3), and *PSMB3* (17q12) that demonstrated focal amplifications in > 10 cancer types (Fig. 2B**-**D).

In total, 3% of the 2,935 genetic variants were found to harbor DNA amplification of PSM genes (*n* = 31) in conjunction with mutations (*n* = 37; BLCA, BRCA, CESC, COADREAD, ESCA, HNSC, LUAD, LUSC, SARC, SKCM, STAD, UCEC) or fusions (*n* = 40; BLCA, BRCA, CESC, CHOL, ESCA, LIHC, LUAD, OV, SARC, SKCM, UCS) in the same patient (Supplementary Tables 1 and 4). Although all 77 co-occurrences of amplification/mutation or amplification/fusion were unique, six patients with BRCA, CHOL, HNSC, LIHC, LUAD, or UCEC harbored two different amplification/mutation (*PSMC2* or *PSMC5*) or amplification/fusion events (*PSMB2* or *PSMD11*) in the same gene or two different genes (*PSMD4* and *PSMG3* in a LUAD sample, and *PSMD11* and *PSMD12* in a BRCA sample). The PSM gene was most commonly the 5’- gene partner (58%), and co-expression between the fusion gene partners was relatively weak (r_s_ <|0.4|). According to Polyphen-2 functional prediction annotation scores, 18/40 amplification/fusion and 17/37 amplification/mutation events were predicted to be possibly damaging (Polyphen-2 scores 0.15 to 1). In contrast, 12/40 amplification/fusion events in *PSMB2*, *PSMB3*, *PSMC4*, *PSMD3*, *PSMD4*, and *PSMD11*, and 12/37 amplification/mutation events in *PSMA6*, *PSMA8*, *PSMB8*, *PSMC2*, *PSMC6*, *PSMD2*, *PSMD3*, and *PSMD4* were more confidently predicted to be damaging (Polyphen-2 scores 0.85 to 1).

Of the 2,935 genetic variants identified in the 49 PSM genes, 2,782 (95%) were classified as potentially deleterious (Supplementary Table 4). Although SIFT and/or Polyphen-2 functional prediction annotation data were not available for 1,233 of the 2,782 (44%) genetic variants, 961 and 900 potentially damaging variants were identified, respectively. Consequently, 721 potentially damaging variants were identified by both databases in 28/32 cancer types and in all PSM genes, except *PSMB10* and *PSMG1-4*. Of the 49 PSM genes, *PSME4* had the highest number of mutations, primarily consisting of missense mutations though other mutations were also identified (*e.g.* nonsense mutation, fusions, amplifications; Fig. 2E). As expected, copy number alterations in the *PSME4* gene such as amplification and deep deletion resulted in over- and underexpression, respectively. However, *PSME4* expression varied in samples harboring missense mutations (Fig. 2F). Although missense mutations spanned the *PSME4* gene, 14 cancer samples (colon adenocarcinoma (COAD, *n* = 2), stomach adenocarcinoma (STAD, *n* = 6), and uterine corpus endometrial carcinoma (UCEC, *n* = 6)) had truncating mutations in a domain at the C-terminal region with unknown function (10 with frameshift deletion in T1805Pfs*69, three with frameshift insertion in T1805Nfs*11, and one sample with missense in *T1805P*; Fig. 2G).

In the breast cancer validation dataset, only *PSMA4* (HER2/ER- subtype, *n* = 2; bilateral breast cancer), *PSMB7* (Luminal B/HER2- subtype, *n* = 1), *PSMD3* (Luminal B/HER2- subtype, *n* = 3; Luminal B/HER2 + subtype, *n* = 1; Basal-like subtype, *n* = 1), and *PSME4* (Luminal B/HER2- subtype, *n* = 2) harbored mutations. DNA amplification was prevalent in 33/39 PSM genes, where five genes (*PSMA7*, *PSMB4*, *PSMD2-4*, *PSMD10*) were amplified in more than 10% of all samples (Supplementary Table 3). These five genes were significantly overexpressed in amplified samples compared to non-amplified breast cancer samples (*P* < 0.0001; t-test). Amplification of *PSMA7*, *PSMB4*, *PSMD4*, and *PSMD10* were identified in the Luminal B, HER2/ER-, and Basal-like subtypes, while *PSMD3* amplification was only found in Luminal B and HER2/ER- samples and *PSMD2* amplification in Luminal B and Basal-like samples. These findings were in agreement with the cBioPortal TCGA dataset. Taken together, these data show that although genetic aberrations were found in all PSM genes, specific PSM genes are hotspots for DNA amplification in certain cancer types.

### Differential gene expression analysis between cancer and normal tissues identifies cancer-related PSM genes

**Fig. 3:**
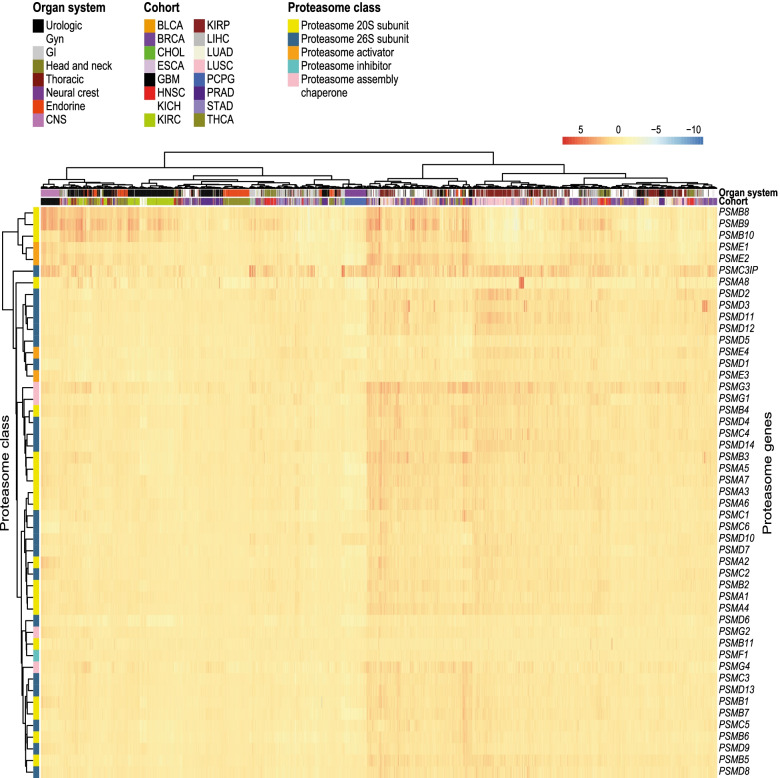
Human proteasome genes frequently displayed overexpression in cancer compared with normal tissue. Heatmap showing relative log2 RSEM gene expression (cancer vs mean normal samples) for the 49 PSM genes in 5,507 TCGA cancer samples representing 16 pan-cancer diseases. Hierarchical clustering was performed with the pheatmap R package (version 1.0.12) using the Manhattan distance metric and Ward’s minimum variance method (Ward.D2)

**Fig. 4:**
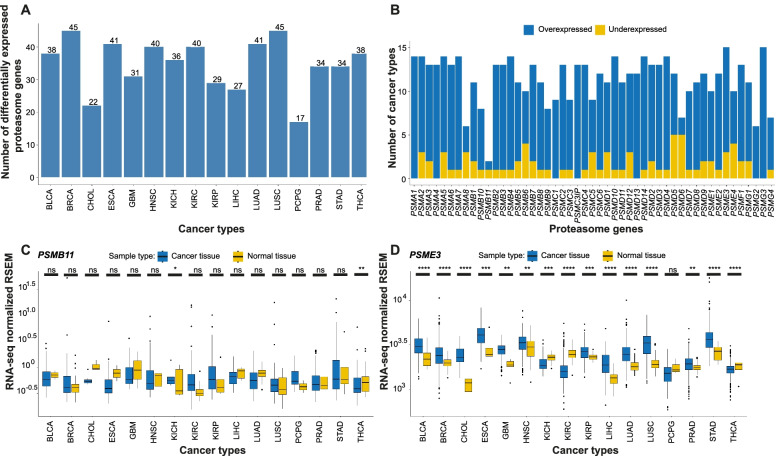
Differentially expressed PSMs between 16 cancer types and corresponding normal tissue. A Bar chart visualizing the number of differentially expressed PSM genes between cancer and normal tissue. BRCA and LUSC showed the highest number of cancer-related PSMs (*n* = 45), whereas only 17 differentially expressed PSMs were identified in PCPG. **B** Bar chart depicting differential PSM gene expression patterns in various cancer types. Overexpression strongly dominated across all cancer types. **C**-**D** Box plot depicting differentially expressed PSMs in cancer and normal tissue. *PSMB11* was found to be differentially expressed in 2/16 cancer types, while *PSME3* was differentially expressed in all except one of the 16 cancer types. The Wilcoxon test was used to calculate statistical significance (Benjamini–Hochberg adjusted p-values) differences in expression (RSEM) between cancer and normal tissue. ns = not significant (*P* > 0.05); *P < 0.05; **P ≤ 0.01; ***P ≤ 0.001; ****P ≤ 0.0001

Differential gene expression analysis was performed in 16/33 cancer types using RNA-seq data from TCGA cancer samples (*n* = 5,507) with corresponding normal tissue (*n* = 627). Expression profiling of 49 PSM genes revealed similar gene expression patterns across the different cancer types, frequently showing overexpression in cancer in comparison with normal tissue (Fig. 3). Interestingly, hierarchical clustering revealed two main clusters of PSM genes, of which one cluster contained five PSM genes (*PSMB8-10* and *PSME1-2*) with high expression in a number of urologic, CNS, and gynecological cancers (Fig. 3). Furthermore, differential expression was found in 35 ± 2 (mean ± SEM, range 17–45) PSM genes per cancer type. Interestingly, 45/49 PSM genes were differentially expressed in the breast invasive carcinoma (BRCA) and lung squamous cell carcinoma (LUSC) cancer types, while only 17/49 PSM genes were differentially expressed in pheochromocytoma and paraganglioma (PCPG; Fig. 4A). Moreover, 11 ± 0.4 (range 2–15) cancer types were associated with each PSM gene. Overexpression of PSM genes was most prevalent across the range of cancer types. For instance, seven PSM genes (*i.e. PSMA1, PSMA4, PSMC1, PSMC3IP, PSMD13, PSMG2-3* (PSM class I/II/V)) were overexpressed in the majority of the 16 cancer types (Fig. 4B). In comparison with the other PSM genes, differential expression of *PSMB11* was relatively uncommon, whereas *PSME3* and *PSMG3* were found to be differentially expressed in virtually all examined cancer forms (15/16 cancer types; Fig. 4C**-**D). Taken together, these findings demonstrate that the vast majority of PSM genes were cancer-related.

### Pearson correlation reveals five clusters of co-expressed PSM genes in cancer

**Fig. 5:**
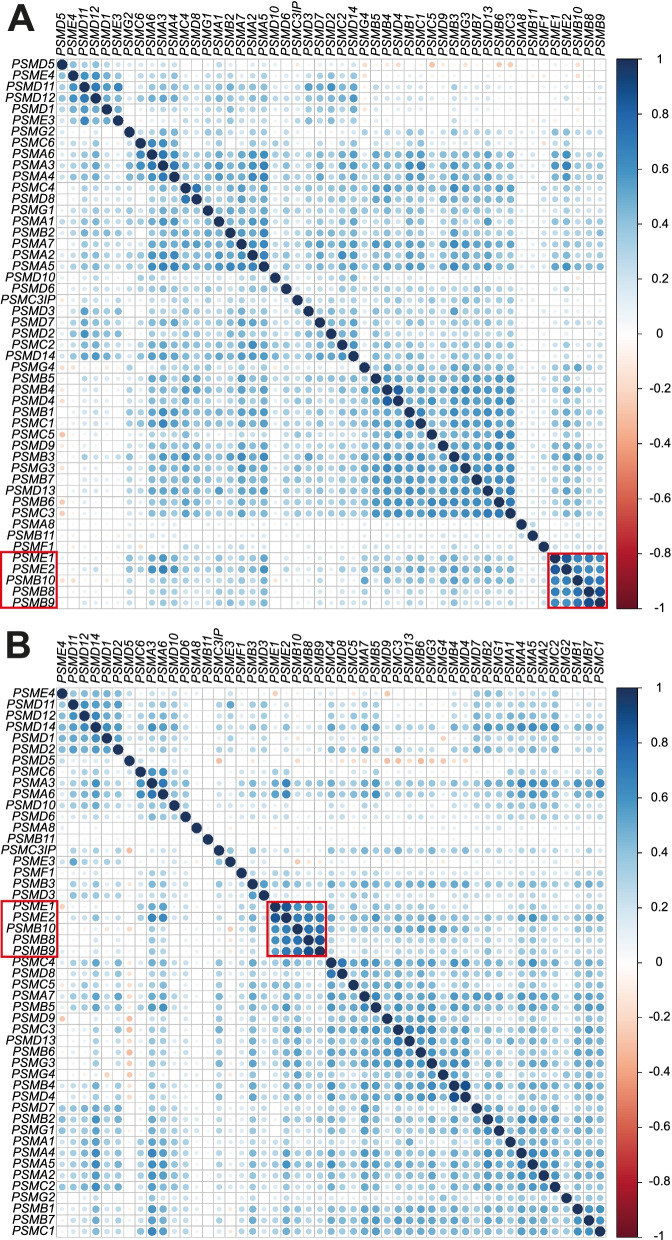
Pairwise Pearson correlation between PSM gene expression in 33 pan-cancer diseases. Correlation matrices for compiled gene expression patterns for (**A**) the 33 pan-cancer diseases and (**B**) BRCA, with genes ordered using hierarchical clustering with Ward’s minimum variance (Ward.D2). Red and blue dots represent negative and positive correlation patterns, respectively. The strength of color and circle size defines correlation pattern between gene pairs using correlation coefficients (*P* < 0.05); blank squares were not statistically significant (*P* > 0.05). PSM genes showing recurrent positive correlation are outlined in red

To assess co-expression of the 49 PSM genes in cancer, pairwise Pearson correlation coefficients (*r*) were calculated for the PSM genes in the 33 cancer types. First, we evaluated overall PSM co-expression patterns in cancer by compiling RNA-seq data for all 33 cancer types. This analysis showed that the majority of co-expressed PSM genes were positively correlated, with at least five gene clusters displaying moderate to strong positive correlation (*r* >|0.4|: 1) *PSMD1, PSMD11-12, PSME3-4, 2) PSMA3-4, PSMA6, PSMC6*, 3) *PSMA2, PSMA5, PSMA7, PSMB2,* 4) *PSMB1, PSMB3-7, PSMC1, PSMC3, PSMC5, PSMD4, PSMD9, PSMD13, PSMG3*, and 5) *PSMB8-10, PSME1-2*; Fig. 5A). In contrast, Pearson correlation coefficients varied between |0.4| and |0.9| for the 33 cancer types. Interestingly, *PSMB8-10* (PSM class I) displayed moderate to strong positive correlation patterns in 31 cancer types (*e.g.* KIRC, LIHC, LUAD). Furthermore, *PSMB8-10* (PSM class I) expression was also strongly correlated with *PSME1-2* (PSM class III) in 27 cancer types, *e.g*. BRCA (Fig. 5B). Consequently, a number of PSM genes belonging to different PSM gene classes were found to be positively correlated, particularly *PSMB8-10*, which are found in the immunoproteasome.

### Multivariable Cox regression analysis shows the prognostic significance of PSM gene expression in cancer

**Fig. 6:**
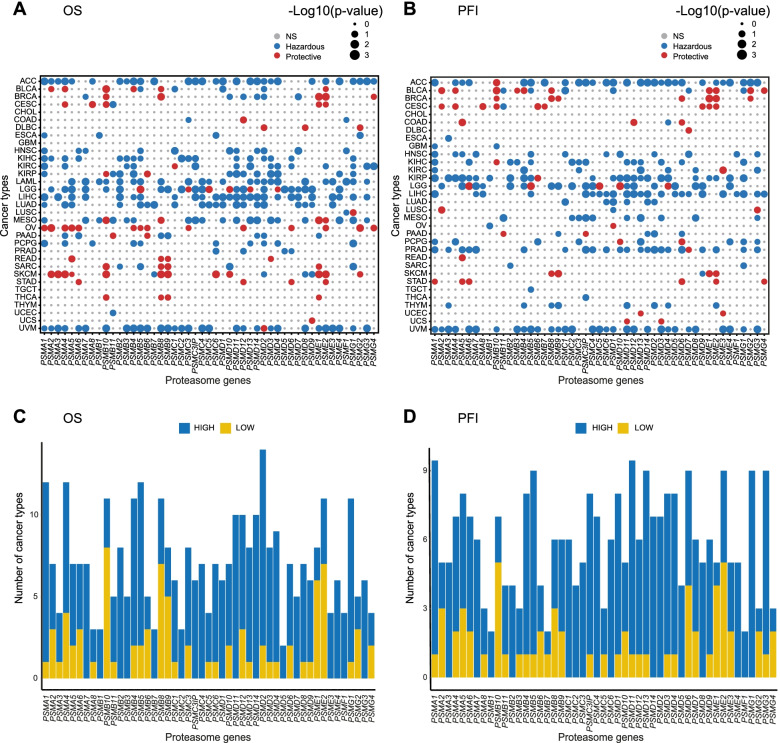
The prognostic relevance of PSM gene expression in different cancer types using overall survival (OS) and progression-free interval (PFI) as clinical endpoints in multivariable Cox regression analysis (adjusted for age and/or tumor grade). **A-B** Dot plots displaying the –log10(p-value) for the multivariable Cox regression analysis between PSM gene expression and OS (**A**) and PFI (**B**). Blue dots indicate a hazardous role for PSM gene expression, while red dots indicate a protective role. NS = not significant (*P* > 0.05). Dot sizes denote –log10(*p*-value); *P* < 0.001 is shown as –log10(*p***-**value) = 3. Due to a lack of clinical data, PFI could not be performed for acute myeloid leukemia (LAML). **C-D** Bar charts illustrating the number of cancer types associated with different expression levels for each prognostic PSM gene. PSM gene expression (high [blue bars, higher than median expression] and low [yellow bars, lower than median expression]) associated with OS (**C**) and PFI (**D**) in different cancer types

To assess the prognostic significance of PSM genes, log2 Fragments Per Kilobase of transcript per Million (FPKM) gene expression (RNA-seq) values were retrieved from the web-based UCSC Xena Browser tool for 10,304 GDC TCGA samples (representing 33 cancer types and 11 pan-cancer body groups; Table 2). Survival analysis was then performed to evaluate the prognostic relevance of the 49 PSM genes in 33 cancer types using overall survival (OS) and progression-free interval (PFI) as clinical endpoints adjusted for covariates (age for 33 cancer types and/or tumor grade for 12 cancer types; Fig. 6A**-**B). Survival analysis for PFI could not be performed for acute myeloid leukemia (LAML) due to a lack of clinical data. In total, age was shown to have an adverse effect on OS in 22/33 cancer types (*e.g.* BRCA, OV, and UVM) and 5/32 cancer types (*e.g.* CESC, LGG, and SKCM) for PFI, but tumor grade only affected prognosis in 3/12 cancer types (*i.e.* HNSC, PAAD, and UCEC) for OS and 4/12 (*e.g.* ESCA, KIRC, and PAAD) for PFI.

**Fig. 7:**
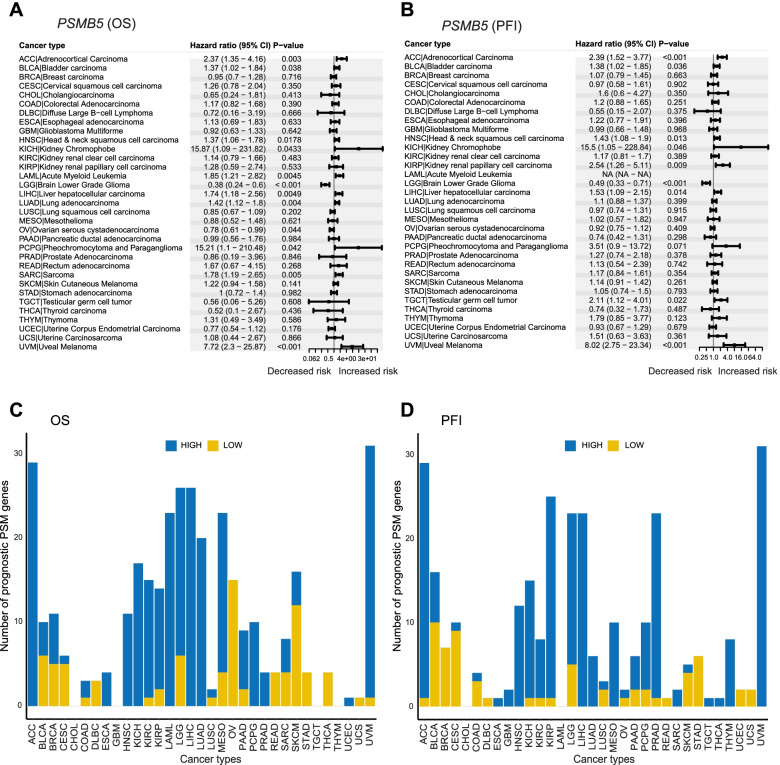
The number of prognostic PSMs associated with high or low expression per cancer type using overall survival (OS) and progression**-**free interval (PFI) as clinical endpoints in multivariable Cox regression analysis (adjusted for age and/or tumor grade). **A-B** Forest plots visualizing the Hazard ratio (HR) for the multivariable Cox regression analysis between high *PSMB5* expression and OS (**A**) and PFI (**B**). HR < 1 shows reduced risk at high *PSMB5* expression (higher than median expression) and HR > 1 illustrates increased risk at high *PSMB5* expression. **C-D** Bar charts visualizing the number of prognostic PSMs associated with each cancer type at high (blue bars, higher than median expression) or low (yellow bars, lower than median expression) expression for OS (**C**) and PFI (**D**)

In total, PSM gene expression (high or low expression) was shown to affect prognosis in 7.1 ± 0.4 (mean ± SEM, range 2–14 (OS)) and 6.0 ± 0.3 (mean ± SEM, range, 2–11 (PFI)) cancer types (Fig. 6C**-**D and Supplementary Fig. 3). Furthermore, PSM genes linked to decreased survival (OS and PFI) were also investigated in ≥ 30% of cancer types. For OS, 12 prognostic PSM genes (*i.e. PSMA1*, *PSMA4*, *PSMB4-5*, *PSMB8*, *PSMB10*, *PSMD2*, *PSMD11-12*, *PSMD14*, *PSME2*, and *PSMG1*; PSM class I/II/III/V) were identified in ≥ 30% of cancer types (Fig. 6C), whereas only two PSM genes (*PSMA1*, *PSMD11*; PSM class I/II) were identified for PFI (Fig. 6D). In addition, *PSMD2* had an impact on prognosis in 42% (14/33) of all cancer types for OS (Supplementary Fig. 4). Interestingly, *PSMB8-10* and *PSME1-2* genes had a significant impact on OS in most cancer types, primarily when underexpressed (Fig. 6C). In contrast, overexpression of *PSMB5*, an important catalytic site in the proteasome, was associated with decreased OS and PFI in 36% and 27% of cancer types, respectively (Figs. 6C**-**D and 7A**-**B).

In contrast, specific cancer types were associated with 10.6 ± 1.6 (range, 0–31 (OS)) and 9.0 ± 1.6 (range, 0–31 (PFI)) prognostic PSM genes (Fig. 7C**-**D). Moreover, specific cancer types were identified where ≥ 50% of PSM genes (up**-** or downregulation) were linked to more unfavorable survival, with overexpression being most common. For OS, four cancer types (*i.e.* ACC (29 genes), LGG (26 genes), LIHC (26 genes), and UVM (31 genes)) were identified (Fig. 7C), and three cancer types (*i.e.* ACC (29 genes), KIRP (25 genes), and UVM (31 genes)) were identified for PFI (Fig. 7D). Interestingly, > 60% of PSM genes (predominantly overexpressed) were associated with both reduced OS and PFI in UVM (Fig. 7C**-**D and Supplementary Fig. 4). Consequently, these results show that PSM gene expression patterns may be an important indicator of prognosis in various cancer types. Compared to the TCGA dataset, similar correlation patterns between PSM gene expression and survival were observed in the breast cancer validation dataset and KM plotter (Supplementary Table 5).

## Discussion

The proteasome is an evolutionarily conserved protein complex that is essential for the maintenance of cellular proteostasis by degrading unneeded and temporary proteins [56]. Therefore, nonfunctional proteasomes lead to severe diseases [3]. In cancer, the proteasome is therefore considered to be a “key player” in tumor progression due to the abnormally high proteasome activity observed in various neoplastic tissues [57]. High proteasome activity is likely due to increased levels of ubiquitinated and/or high expression of proteasome subunits [57]. Here, we performed a comprehensive pan**-**cancer study of PSM genes using a large public dataset from The Cancer Genome Atlas and the cBioPortal web**-**based online tool to investigate the effect of genetic alterations and subsequent changes in PSM gene expression on prognosis. The study was limited by the lack of large datasets (similar to The Cancer Genome Atlas dataset) to validate our findings and the inclusion of metastatic lesions in the SKCM and THCA datasets; the results for SKCM in particular should be interpreted with this in mind. Nevertheless, we were able to reveal a connection between frequent overexpression of specific PSM genes and adverse patient clinical outcome in several cancer types. These findings suggest that a number of PSM genes can be important prognostic and therapeutic markers for cancer.

Amplification events and subsequent overexpression of target genes are relatively common in cancer genomes [58]. In particular, cancer drivers are frequently found in genomic regions of focal amplification [59, 60]. Although genetic alterations were found to occur in all PSM genes, alteration frequencies varied in the different cancer types. In general, two different patterns of DNA amplification were observed, i.e. focal amplification of specific PSM genes (e.g. *PSMB3*, *PSMB4*, and *PSMD4*) in thoracic and gynecologic organ systems and focal amplification in conjunction with either mutations or fusions of the same PSM gene. Although uncommon, these findings indicate that specific PSM genes are targeted by more than one molecular mechanism for activation. These focal amplification events may possibly be due to proximity to a mutation hotspot region. Furthermore, co**-**amplification of PSM genes located in close proximity to one another (e.g. *PSMB5* and *PSMB11*, 14q11.2; *PSMB4* and *PSMD4*, 1q21.3; *PSMB8* and *PSMB9*, 6p21.32) or known cancer drivers (e.g. co**-**amplification of *ERBB2* and *PSMB3*) were also frequently amplified together. Intriguingly, amplification of *PSMB3*, *PSMB4*, and *PSMD4* have also been observed in breast**-** and ovarian cancer [61–63].

However, mutation events in PSM genes were relatively rare in cancer, which was also observed in the breast cancer validation dataset where only four PSM genes (*PSMA4*, *PSMB7*, *PSMD3*, and *PSME4*) harbored mutations. These findings indicate that mutations could cause loss of proteasome function thereby causing cell death. Although focal DNA amplification of PSM genes was found to have a significant effect on the expression levels of individual PSM genes, it could not account for the global overexpression observed in most cancer types due to its infrequency. This indicates that other molecular mechanisms (*e.g.* DNA methylation, histone modification or transcription regulation) contribute to the aberrant PSM gene expression patterns shown in cancer. For example, the NRF1 and NRF2 transcription factors are known to induce transcription of PSM genes during different types of cellular stress. Recent studies have shown that inhibition of the β2 proteasome site leads to the aggregation of NRF1, thereby suppressing proteasome gene expression and the production of new proteasomes [3, 34, 38, 64]. Consequently, the elevated PSM gene expression patterns and hence high proteasome activity observed in cancer suggests an underlying dependency on the ubiquitin–proteasome system and thereby therapeutic vulnerability to proteasome inhibition.

To further evaluate the significance of PSM expression levels in cancer, we performed differential expression analysis of the PSM genes in cancer and corresponding normal tissue. This analysis showed that most PSM genes, especially *PSME3* and *PSMG3*, were differentially expressed (frequently overexpressed) relative to normal tissue, further highlighting the importance of the proteasome in cancer development and progression. As *PSME3* and *PSMG3* are involved in proteasome activation and assembly, evaluation of their expression levels in cancer could be clinically relevant. Unfortunately, differential expression analysis was only performed on 16/33 cancer types due to the lack of or limited number of corresponding normal tissue samples. Nevertheless, high *PSME3* expression has been previously associated with worse survival in colorectal cancer; our data confirm that *PSME3* may also be important as a prognostic and predictive biomarker for other types of cancer [65].

Pearson correlation analysis revealed that co-expression of most PSM genes were positively correlated. In general, cancer was shown to co-express (strong positive correlation) at least five PSM gene clusters (1) *PSMD1, PSMD11-12, PSME3-4, 2) PSMA3-4, PSMA6, PSMC6*, 3) *PSMA2, PSMA5, PSMA7, PSMB2,* 4) *PSMB1, PSMB3-7, PSMC1, PSMC3, PSMC5, PSMD4, PSMD9, PSMD13, PSMG3*, and 5) *PSMB8-10, PSME1-2*. These findings demonstrate that co-expression of PSM subunits, activators (*PSME1-4*; facilitates access to the proteasome complex [66]), and assembly genes (*PSMG3*; assembly chaperone that allows for efficient proteasome assembly [67]) are required to ensure high-fidelity organization and assembly of the proteasome. The diverse mutation profiles, expression patterns, and co-expression patterns shown in the different cancer types may be due to a number of factors, including proteasome structural diversity in different tissues and the need for an assortment of various proteasome subunits (i.e. immunoproteasome, *PSMB8-10*), as well as, differences in proteasome regulation (i.e. proteasome activators, *PSME1-2*) [68–74]. The expression of *PSMB8-10* (class I) was nevertheless shown to be highly correlated in 31 cancer types, with an association between high *PSMB8-10* expression and better survival. These findings are not particularly surprising, as *PSMB8-10* are the catalytic subunits in the immunoproteasome, which plays a pivotal role in the immune system [75].

Survival analysis revealed 12 PSM genes with prognostic potential (*PSMA1*, *PSMA4*, *PSMB4-5*, *PSMB8*, *PSMB10*, *PSMD2*, *PSMD11-12*, *PSMD14*, *PSME2*, and *PSMG1*; PSM class I/II/III/V) for OS and two PSM genes (*PSMA1*, *PSMD11*; PSM class I/II) for PFI. Recently, high expression of several of these PSM genes (e.g. *PSMA1*, *PSMB4*, and *PSMD2*) has been correlated with poor prognosis in a number of cancer types, including breast-, lung-, and gastric cancer [76–78]. In the validation dataset and KM plotter, these PSM genes were also found to be of prognostic value. Notably, *PSMA1* and *PSMD11* were associated with both OS and PFI. These findings indicate that *PSMA1* and *PSMD11* may be useful biomarkers for the early detection of relapse, whereas patient samples expressing aberrant expression patterns of the 12 OS-related PSM genes may warrant more aggressive treatment regimens. Although overexpression of the PSM genes was most frequently associated with prognosis, underexpression of *PSMB8-10* had a major impact on prognosis in several cancer types. This is consistent with recent studies revealing that high expression of the immunoproteasome is associated with better survival in breast cancer [79]. Intriguingly, overexpression of one of the three proteasome catalytic sites, *PSMB5*, had an adverse effect on prognosis in 12 (OS) and 9 (PFI) of the studied cancer types. The prognostic significance of *PSMB5* is consistent with a previous study that established a link between high *PSMB5* expression and enhanced tumor progression in breast cancer [80]. *PSMB5* is also the main target for most clinically relevant proteasome inhibitors, further highlighting its importance for proteasome function and cell survival. We also identified specific cancer types where the majority of PSM genes had an impact on prognosis. Therefore, patients with ACC, LGG, LIHC, and UVM showing consistently elevated proteasome activity due to PSM gene overexpression might benefit from proteasome inhibitor-based treatment or targeted treatment with inhibitors for individual PSM genes. However, several cancer types can be characterized into histological subtypes due to heterogeneity. Consequently, it may be necessary to perform an in-depth analysis of specific cancer types to identify subtypes that may benefit from proteasome inhibition.

In conclusion, the comprehensive pan-cancer analysis presented here demonstrated that several PSM genes (*e.g. PSMA1*, *PSMB4-5*, *PSMB8-10*, *PSMD2*, *PSMD4*, *PSMD11*, *PSME1-3*, and *PSMG3*) may be putative biomarkers for determining prognosis and choice of treatment for different cancer types. However, the proteasome is a complex of several PSM proteins and crosstalk between different PSMs is inherent for proteasome activity. Therefore, further studies are needed to identify a panel(s) of up- or down-regulated PSMs that are associated with patients at-risk of cancer-related death and recurrence, thereby potentially improving the survival of cancer patients.

## Supplementary Information

**Additional file 1: Supplementary Figure 1.** OncoPrint conducted by cBioPortal displaying genetic alteration frequency of proteasome genes.

**Additional file 2: Supplementary Figure 2.** Stacked bar chart depicting the number of amplifications per PSM gene in the 32 cancer types. **Supplementary Figure 3.** PSM focal amplification and gene expression associated with patient survival (OS and PFI). **Supplementary Figures 4.** Forest plots depicting multivariable Cox regression analysis and prognostic relevance (OS and PFI) PSM gene expression patterns in UVM patients and PSM gene (PSMA1 and PSMD2) expression patterns and survival risk in 33 cancer types. HR <1 depicts the association between high PSM gene expression and decreased risk of survival, whereas HR >1 illustrates the association between high PSM gene expression and increased risk of survival.

**Additional file 3: Supplementary Table 1.** Amplification of the 49 human proteasome gene family members.

**Additional file 4: Supplementary Table 2.** Mutation signature of proteasome genes in 23 breast invasive carcinomas.

**Additional file 5: Supplementary Table 3.** Amplification and deletion of the 49 human proteasome gene family members in breast cancer.

**Additional file 6. Supplementary Table 4.** Mutations in the 49 PSM genes across TCGA samples.

**Additional file 7: Supplementary Table 5.** External validation of survival analysis (Overall survival; OS) for the 49 human proteasome gene family members.

## Data Availability

The data for the breast cancer validation cohort used in this study have already been deposited in Gene Expression Omnibus (accession GSE97293), as stated in our previous publication [48]. The databases referenced in the methods section of this article are all open access.
