# Supplementary material for: Pan-cancer analysis of genomic and transcriptomic data reveals the prognostic relevance of human proteasome genes in different cancer types
Source: BMC Cancer. 2022 Sep 19;22:993. doi: 10.1186/s12885-022-10079-4 (PMC9484138; doi:10.1186/s12885-022-10079-4)
Supplement: Supplementary file 1 — Additional file 1: Supplementary Figure 1. OncoPrint conducted by cBioPortal displaying genetic alteration frequency of proteasome genes. [file 12885_2022_10079_MOESM1_ESM.pdf]

Supplementary figure 1

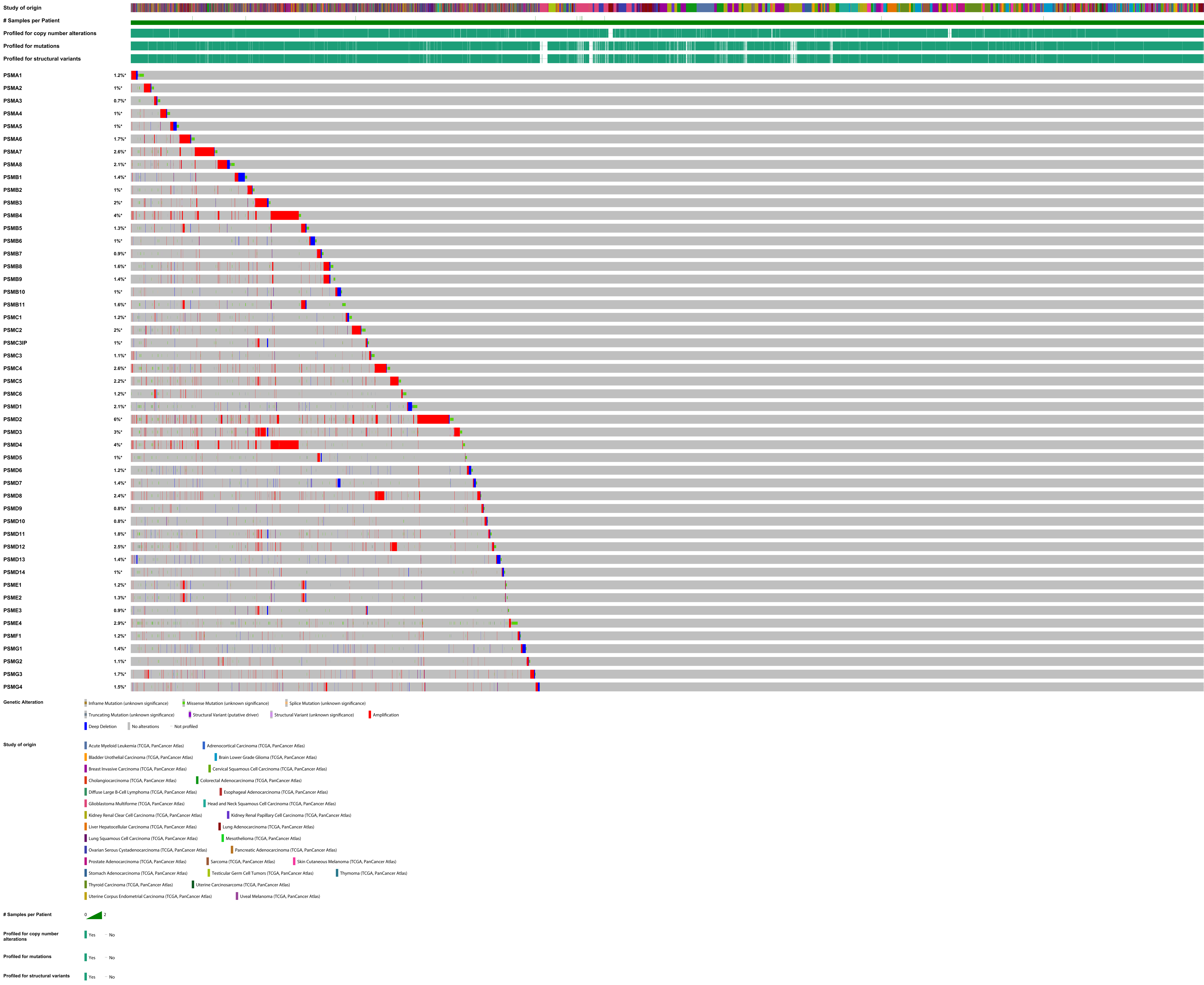

Supplementary Figure 1. OncoPrint conducted by cBioPortal displaying genetic alteration frequency of proteasome genes
