# Supplementary material for: Pan-cancer analysis of genomic and transcriptomic data reveals the prognostic relevance of human proteasome genes in different cancer types
Source: BMC Cancer. 2022 Sep 19;22:993. doi: 10.1186/s12885-022-10079-4 (PMC9484138; doi:10.1186/s12885-022-10079-4)
Supplement: Supplementary file 2 — Additional file 2: Supplementary Figure 2. Stacked bar chart depicting the number of amplifications per PSM gene in the 32 cancer types. Supplementary Figure 3. PSM focal amplification and gene expression associated with patient survival (OS and PFI). Supplementary Figures 4. Forest plots depicting multivariable Cox regression analysis and prognostic relevance (OS and PFI) PSM gene expression patterns in UVM patients and PSM gene (PSMA1 and PSMD2) expression patterns and survival risk in 33 cancer types. HR <1 depicts the association between high PSM gene expression and decreased risk of survival, whereas HR >1 illustrates the association between high PSM gene expression and increased risk of survival. [file 12885_2022_10079_MOESM2_ESM.pdf]

Supplementary Figure 2.

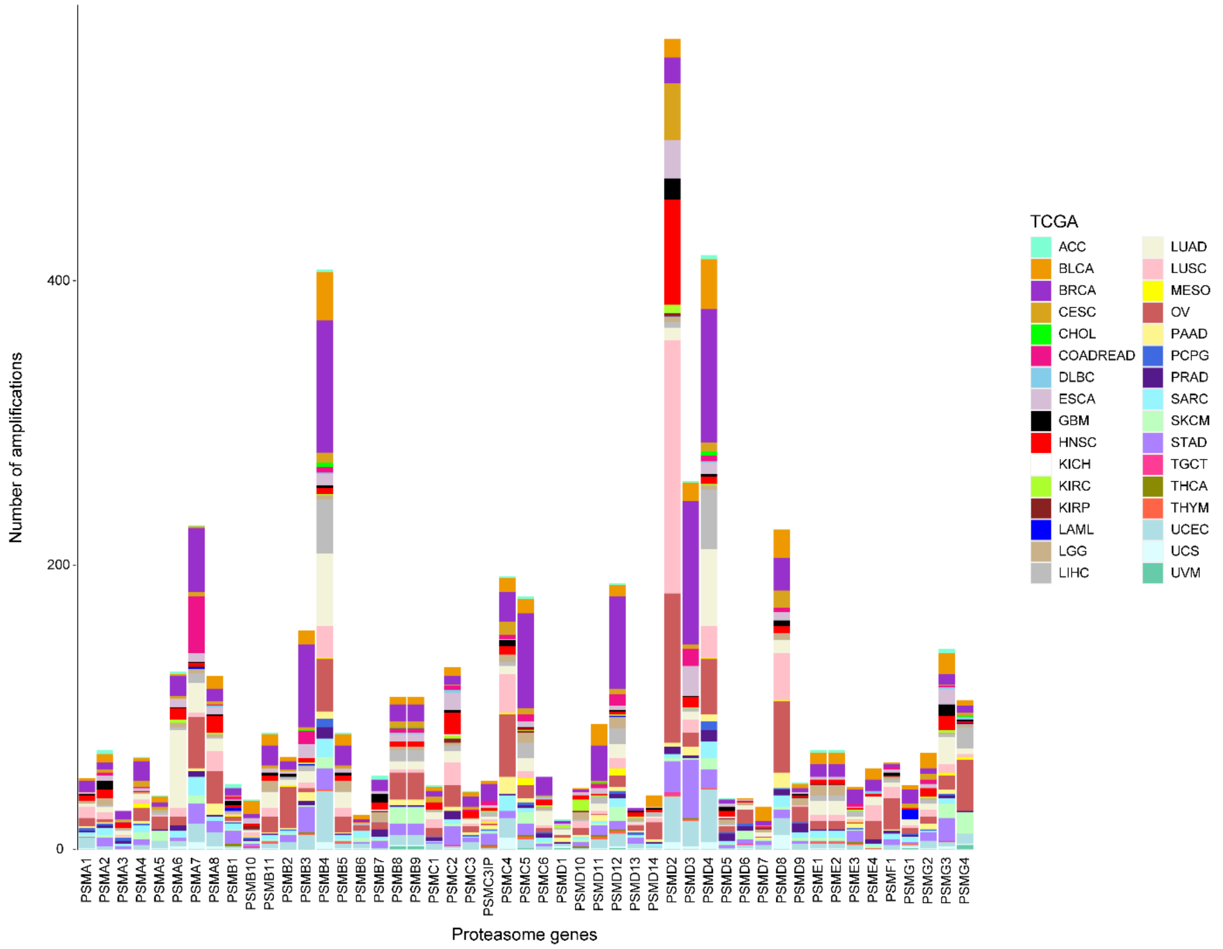

Supplementary Figure 2. Stacked bar chart depicting the number of amplifications per PSM gene in the 32 cancer types.

Supplementary Figure 3.

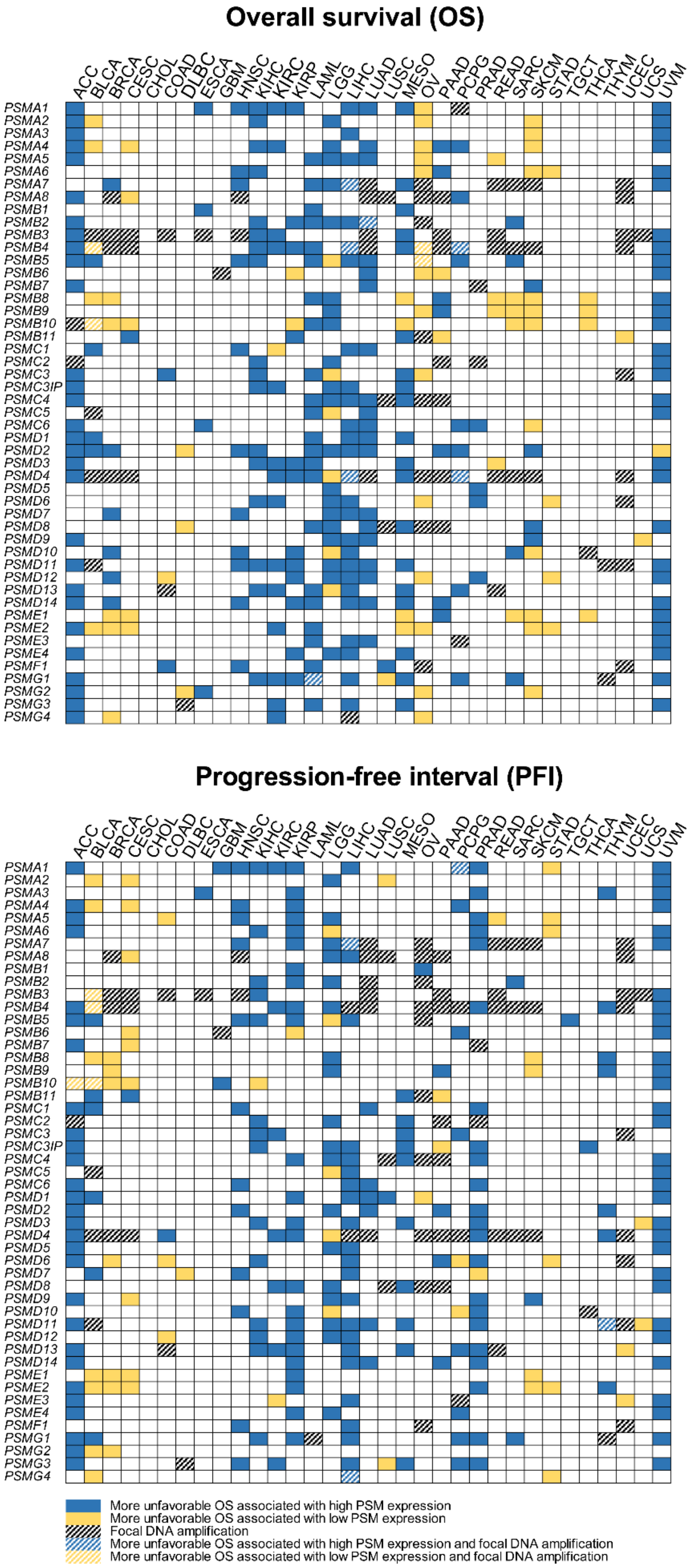

Supplementary Figure 3. PSM focal amplification and gene expression associated with patient survival (OS and PFI).

Supplementary Figure 4.

PSMA1 (PFI)

Cancer type	Hazard ratio (95% CI)	P−value
ACC|Adrenocortical Carcinoma	2.97 (1.51 − 5.86)	0.002
BLCA|Bladder carcinoma	0.81 (0.57 − 1.13)	0.216
BRCA|Breast carcinoma	0.9 (0.62 − 1.31)	0.581
CESC|Cervical squamous cell carcinoma	0.82 (0.42 − 1.42)	0.472
CHOL|Cholangiocarcinoma	1.45 (0.31 − 6.74)	0.633
COAD|Colorectal Adenocarcinoma	0.91 (0.62 − 1.34)	0.648
DLBC|Diffuse Large B−cell Lymphoma	2.87 (0.38 − 21.55)	0.306
ESCA|Esophageal adenocarcinoma	0.88 (0.53 − 1.47)	0.633
GBM|Glioblastoma Multiforme	1.51 (1 − 2.26)	0.048
HNSC|Head & neck squamous cell carcinoma	1.47 (1.11 − 1.96)	0.008
KICH|Kidney Chromophobe	44.16 (2.77 − 705.09)	0.007
KIRC|Kidney renal clear cell carcinoma	1.77 (1.08 − 2.9)	0.024
KIRP|Kidney renal papillary cell carcinoma	3.94 (1.85 − 8.35)	<0.001
LAML|Acute Myeloid Leukemia	NA (NA − NA)	
LGG|Brain Lower Grade Glioma	1.28 (0.81 − 2.05)	0.293
LIHC|Liver hepatocellular carcinoma	2.27 (1.55 − 3.33)	<0.001
LUAD|Lung adenocarcinoma	1.2 (0.87 − 1.66)	0.271
LUSC|Lung squamous cell carcinoma	1.16 (0.88 − 1.53)	0.305
MESO|Mesothelioma	2.45 (1 − 6.02)	0.051
OV|Ovarian serous cystadenocarcinoma	0.91 (0.72 − 1.17)	0.467
PAAD|Pancreatic ductal adenocarcinoma	1.52 (0.89 − 2.58)	0.126
PCPG|Pheochromocytoma and Paraganglioma	2.91 (1.17 − 7.22)	0.022
PRAD|Prostate Adenocarcinoma	2.76 (1.36 − 5.62)	0.005
READ|Rectum adenocarcinoma	0.6 (0.3 − 1.21)	0.156
SARC|Sarcoma	1 (0.71 − 1.42)	0.99
SKCM|Skin Cutaneous Melanoma	0.81 (0.62 − 1.05)	0.113
STAD|Stomach adenocarcinoma	0.7 (0.49 − 0.99)	0.046
TGCT|Testicular germ cell tumor	1.2 (0.65 − 2.22)	0.555
THCA|Thyroid carcinoma	0.71 (0.26 − 1.92)	0.495
THYM|Thymoma	4.24 (0.94 − 19.04)	0.06
UCEC|Uterine Corpus Endometrial Carcinoma	0.92 (0.65 − 1.29)	0.617
UCS|Uterine Carcinosarcoma	1.57 (0.69 − 3.58)	0.283
UVM|Uveal Melanoma	3.83 (1.36 − 10.75)	0.011

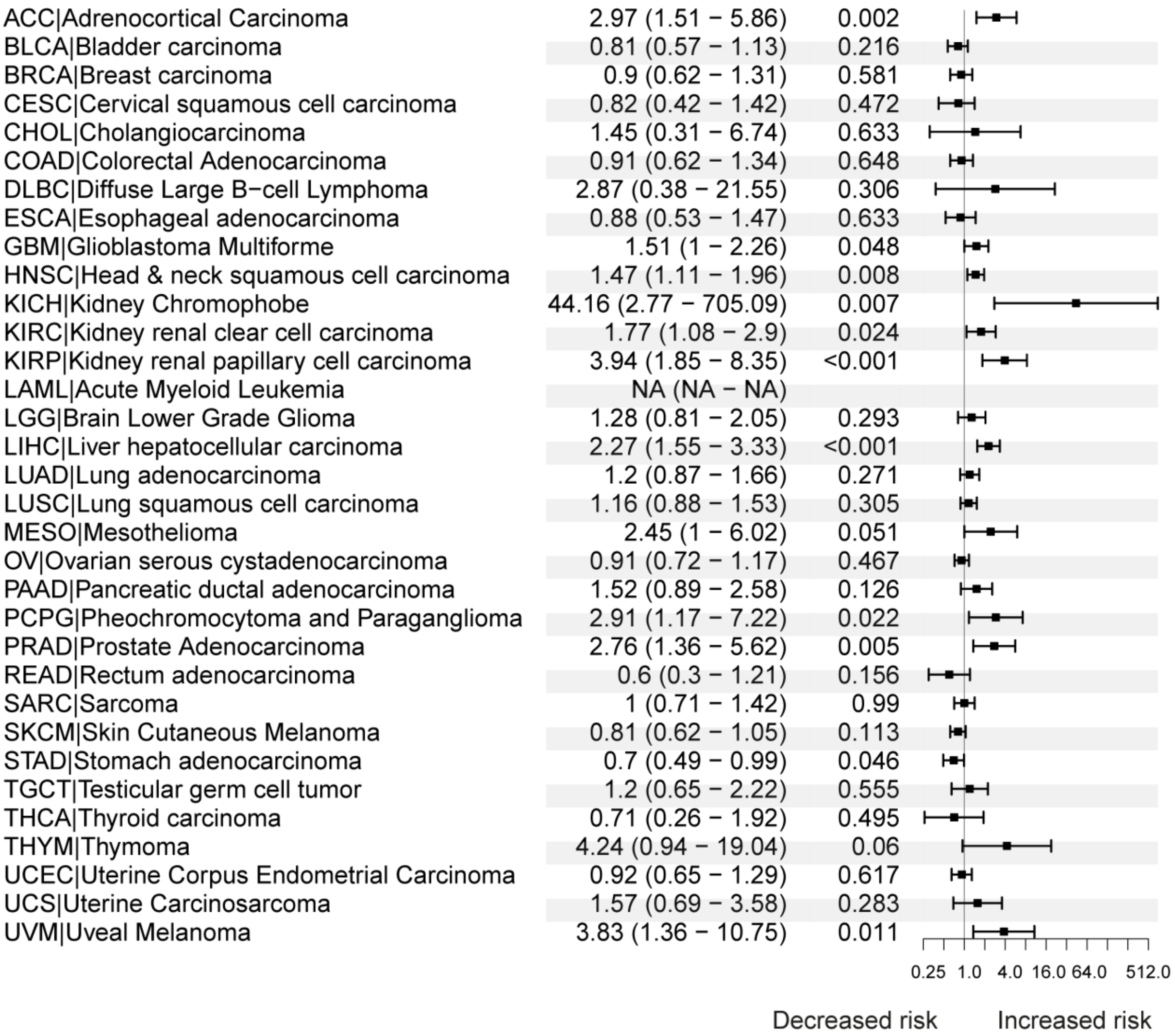

PSMD2 (OS)

Cancer type	Hazard ratio (95% CI)	P−value
ACC|Adrenocortical Carcinoma	2.22 (1.2 − 4.12)	0.011
BLCA|Bladder carcinoma	1.33 (1.04 − 1.69)	0.024
BRCA|Breast carcinoma	1.39 (1.05 − 1.85)	0.023
CESC|Cervical squamous cell carcinoma	0.91 (0.61 − 1.35)	0.628
CHOL|Cholangiocarcinoma	1.29 (0.38 − 4.36)	0.677
COAD|Colorectal Adenocarcinoma	1.34 (0.87 − 2.08)	0.186
DLBC|Diffuse Large B−cell Lymphoma	0.08 (0.01 − 0.56)	0.011
ESCA|Esophageal adenocarcinoma	0.98 (0.64 − 1.48)	0.906
GBM|Glioblastoma Multiforme	1 (0.61 − 1.64)	0.991
HNSC|Head & neck squamous cell carcinoma	1.41 (1.1 − 1.8)	0.007
KICH|Kidney Chromophobe	15.6 (1.14 − 214.29)	0.04
KIRC|Kidney renal clear cell carcinoma	1.39 (0.93 − 2.08)	0.106
KIRP|Kidney renal papillary cell carcinoma	1.65 (0.84 − 3.25)	0.143
LAML|Acute Myeloid Leukemia	2.39 (1.18 − 4.87)	0.016
LGG|Brain Lower Grade Glioma	2.24 (1.31 − 3.84)	0.003
LIHC|Liver hepatocellular carcinoma	1.88 (1.36 − 2.61)	<0.001
LUAD|Lung adenocarcinoma	1.61 (1.27 − 2.05)	<0.001
LUSC|Lung squamous cell carcinoma	0.96 (0.8 − 1.16)	0.666
MESO|Mesothelioma	1.54 (0.85 − 2.77)	0.153
OV|Ovarian serous cystadenocarcinoma	0.92 (0.72 − 1.19)	0.536
PAAD|Pancreatic ductal adenocarcinoma	1.72 (1.06 − 2.8)	0.028
PCPG|Pheochromocytoma and Paraganglioma	5.68 (1.1 − 29.46)	0.039
PRAD|Prostate Adenocarcinoma	2.46 (0.48 − 12.58)	0.281
READ|Rectum adenocarcinoma	0.56 (0.24 − 1.3)	0.175
SARC|Sarcoma	1.07 (0.77 − 1.48)	0.7
SKCM|Skin Cutaneous Melanoma	1.35 (1.02 − 1.78)	0.033
STAD|Stomach adenocarcinoma	1.02 (0.73 − 1.42)	0.898
TGCT|Testicular germ cell tumor	1.54 (0.17 − 14.32)	0.702
THCA|Thyroid carcinoma	0.57 (0.1 − 3.4)	0.539
THYM|Thymoma	1.7 (0.33 − 8.83)	0.529
UCEC|Uterine Corpus Endometrial Carcinoma	0.87 (0.61 − 1.23)	0.428
UCS|Uterine Carcinosarcoma	0.71 (0.35 − 1.46)	0.358
UVM|Uveal Melanoma	0.28 (0.1 − 0.76)	0.013

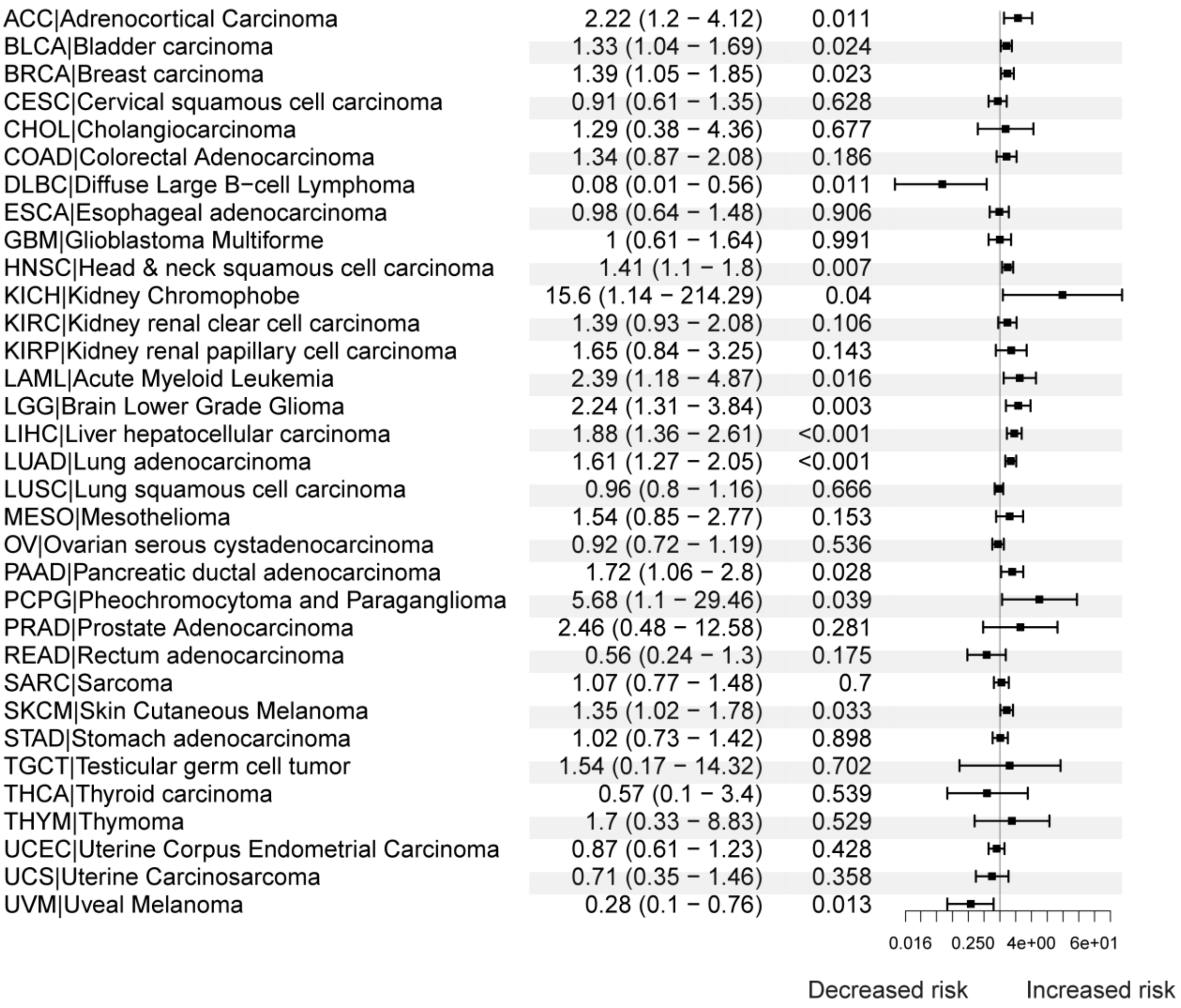

UVM (OS)

PSM gene	Hazard ratio (95% CI)	P−value
PSMA1	3.24 (1.04 − 10.14)	0.043
PSMA2	3.29 (1.05 − 10.38)	0.042
PSMA3	2.52 (1.39 − 4.57)	0.002
PSMA4	2.17 (1.18 − 4)	0.013
PSMA5	1.9 (0.91 − 3.96)	0.087
PSMA6	3.09 (1.29 − 7.42)	0.011
PSMA7	2.96 (1.46 − 5.99)	0.003
PSMA8	0.05 (0.01 − 433.84)	0.516
PSMB1	2.32 (0.98 − 5.49)	0.056
PSMB10	2.21 (1.42 − 3.42)	<0.001
PSMB11	NA (NA − NA)	
PSMB2	1.46 (0.64 − 3.33)	0.364
PSMB3	6.84 (2.76 − 16.98)	<0.001
PSMB4	20.63 (4.35 − 97.96)	<0.001
PSMB5	7.72 (2.3 − 25.87)	<0.001
PSMB6	5.44 (1.91 − 15.51)	0.002
PSMB7	1.44 (0.56 − 3.69)	0.453
PSMB8	1.75 (1.25 − 2.45)	0.001
PSMB9	1.48 (1.19 − 1.84)	<0.001
PSMC1	3.1 (1.06 − 9.04)	0.038
PSMC2	3.66 (1.49 − 9.01)	0.005
PSMC3	6.03 (2.01 − 18.14)	0.001
PSMC3IP	0.7 (0.15 − 3.25)	0.649
PSMC4	7.88 (2.82 − 22.03)	<0.001
PSMC5	7.79 (2.7 − 22.41)	<0.001
PSMC6	1.56 (0.83 − 2.94)	0.165
PSMD1	1.78 (0.74 − 4.3)	0.198
PSMD10	0.95 (0.47 − 1.92)	0.892
PSMD11	5.04 (1.74 − 14.57)	0.003
PSMD12	2.18 (1.1 − 4.31)	0.025
PSMD13	3.26 (0.84 − 12.67)	0.089
PSMD14	2.04 (1.12 − 3.73)	0.02
PSMD2	0.28 (0.1 − 0.76)	0.013
PSMD3	4.33 (1.05 − 17.87)	0.043
PSMD4	14.38 (2.56 − 80.7)	0.002
PSMD5	1.55 (0.66 − 3.67)	0.313
PSMD6	0.42 (0.15 − 1.16)	0.094
PSMD7	2.01 (0.97 − 4.15)	0.06
PSMD8	8.25 (2.2 − 30.96)	0.002
PSMD9	0.85 (0.13 − 5.51)	0.866
PSME1	2.36 (1.2 − 4.65)	0.013
PSME2	2.28 (1.4 − 3.72)	<0.001
PSME3	6.96 (2.09 − 23.19)	0.002
PSME4	2.62 (1.42 − 4.85)	0.002
PSMF1	0.96 (0.26 − 3.63)	0.957
PSMG1	3 (1.11 − 8.1)	0.03
PSMG2	2.57 (0.83 − 7.93)	0.1
PSMG3	5.88 (2.63 − 13.16)	<0.001
PSMG4	0.55 (0.16 − 1.9)	0.341

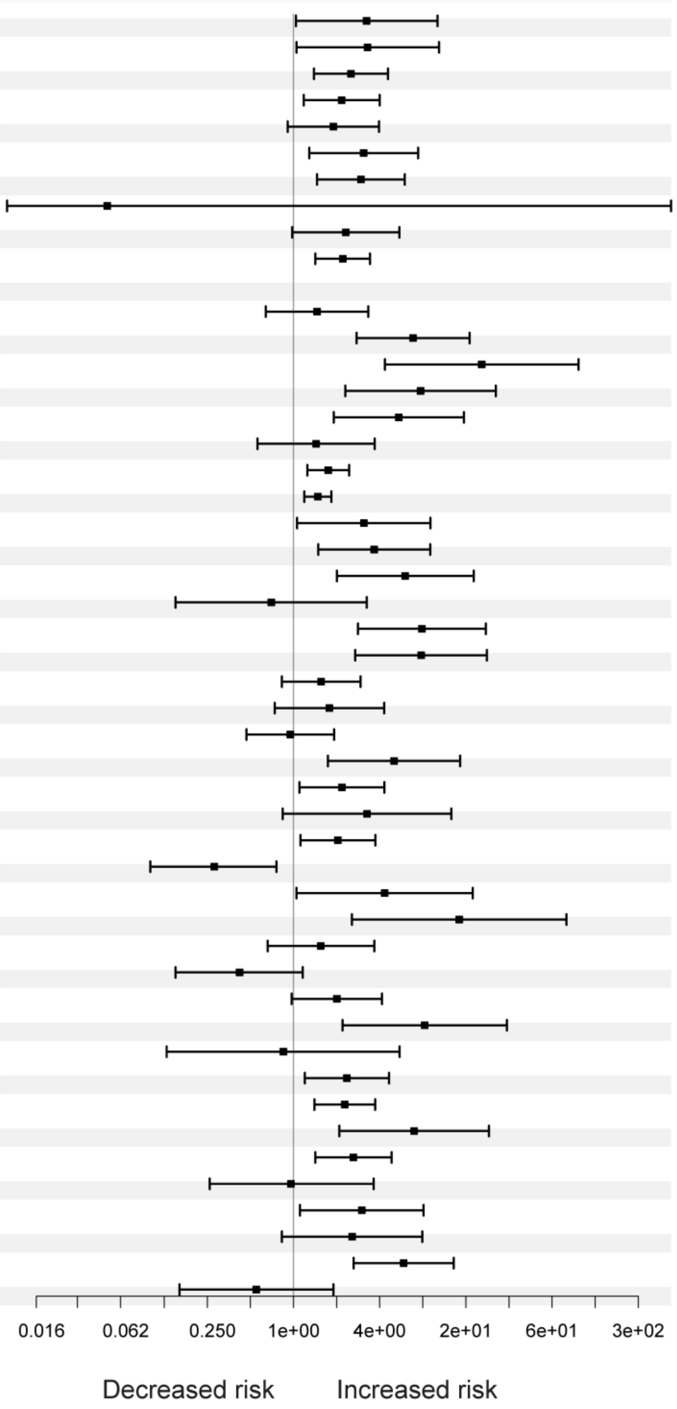

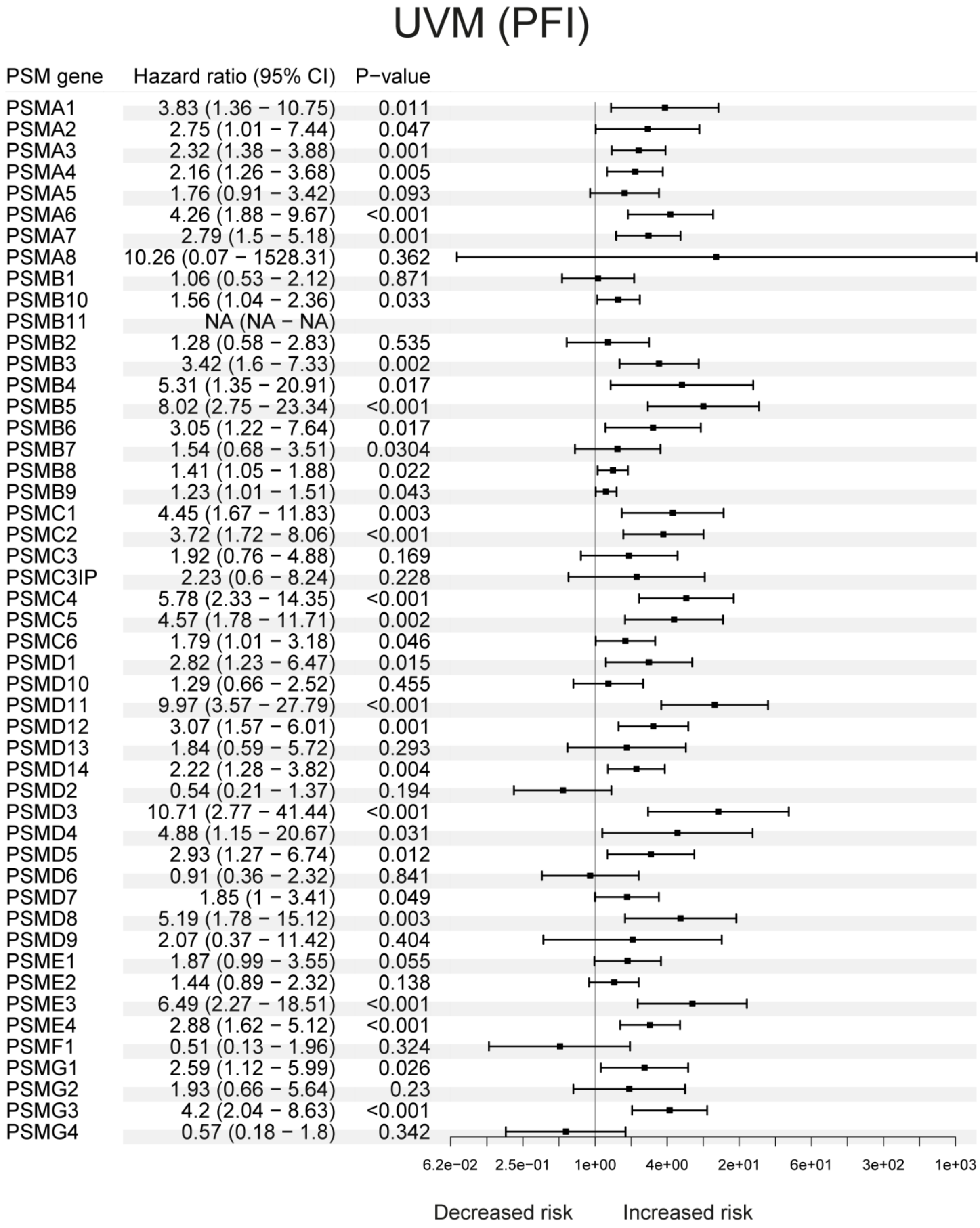

Supplementary Figures 4. Forest plots depicting multivariable Cox regression analysis and prognostic relevance (OS and PFI). PSM gene expression patterns in UVM patients and PSM gene (*PSMA1* and *PSMD2*) expression patterns and survival risk in 33 cancer types. HR <1 depicts the association between high PSM gene expression and decreased risk of survival, whereas HR >1 illustrates the association between high PSM gene expression and increased risk of survival.
